# Carriage of antibiotic-resistant Gram-negative bacteria after discontinuation of selective decontamination of the digestive tract (SDD) or selective oropharyngeal decontamination (SOD)

**DOI:** 10.1186/s13054-018-2170-2

**Published:** 2018-09-29

**Authors:** E. de Jonge, R. B. P. de Wilde, N. P. Juffermans, E. A. N. Oostdijk, A. T. Bernards, E. H. R. van Essen, E. J. Kuijper, C. E. Visser, J. Kesecioglu, M. J. M. Bonten

**Affiliations:** 1 Department of Intensive Care, Leiden University Medical Center, B4-32, Albinusdreef 2, 2300 RC Leiden, The Netherlands; 2 Department of Medical Microbiology, Leiden University Medical Center, Leiden, The Netherlands; 3 Department of Intensive Care, Academic Medical Center, Amsterdam, The Netherlands; 4 Department of Medical Microbiology, Academic Medical Center, Amsterdam, The Netherlands; 5 Department of Intensive Care, University Medical Center, Utrecht, The Netherlands; 6 Department of Medical Microbiology, University Medical Center, Utrecht, The Netherlands

**Keywords:** Selective digestive tract decontamination, Selective oropharyngeal decontamination, Intensive care, Decolonization, Antibiotic resistance, Gram-negative bacteria

## Abstract

**Background:**

Selective decontamination of the digestive tract (SDD) and selective oropharyngeal decontamination (SOD) reduce colonization with antibiotic-resistant Gram-negative bacteria (ARGNB), incidence of nosocomial infections and improve survival in ICU patients. The effect on bacterial gut colonization might be caused by growth suppression by antibiotics during SDD/SOD. We investigated intestinal colonization with ARGNB after discharge from ICU and discontinuation of SDD or SOD.

**Methods:**

We performed a prospective, observational follow-up study in regular hospital wards of three teaching hospitals in the Netherlands in patients discharged from the ICU, who were participating in a cluster randomized trial comparing SDD with SOD. We determined rectal carriage with ARGNB at ICU discharge (time (*T*) = 0) and 3, 6 and 10 days after discharge. The primary endpoint was time to first colonization with ARGNB that was not present at *T* = 0. Bacteria that are intrinsically resistant to antibiotics were not included in the primary analysis, but were included in post-hoc analysis.

**Results:**

Of 1370 patients screened for inclusion, 996 patients had samples at *T* = 0 (507 after SDD and 489 after SOD). At ICU discharge, the prevalence of intestinal carriage with any ARGNB was 22/507 (4.3%) after SDD and 87/489 (17.8%) after SOD (*p* < 0.0001): 426 (SDD) and 409 (SOD) patients had at least one follow-up sample for analysis. The hazard rate for acquiring carriage of ARGNB after discontinuation of SDD, compared to SOD, in the ICU was 0.61 (95% CI 0.40–0.91, *p* = 0.02), and cumulative risks of acquisition of at least one ARGNB until day 10 were 13% (SDD) and 18% (SOD). At day 10 after ICU discharge, the prevalence of intestinal carriage with ARGNB was 11.3% (26/230 patients) after SDD and 12.5% (28/224 patients) after SOD (*p* = 0.7). In post-hoc analysis of all ARGNB, including intrinsically resistant bacteria, colonization at ICU discharge was lower after SDD (4.9 vs. 22.3%, *p* < 0.0001), but acquisition rates after ICU discharge were similar in both groups.

**Conclusions:**

Intestinal carriage at ICU discharge and the acquisition rate of ARGNB after ICU discharge are lower after SDD than after SOD. The prevalence of intestinal carriage with ARGNB at 10 days after ICU discharge was comparable in both groups, suggesting rapid clearance of ARGNB from the gut after ICU discharge.

**Trial registration:**

Netherlands Trial Registry, NTR3311. Registered on 28 february 2012.

**Electronic supplementary material:**

The online version of this article (10.1186/s13054-018-2170-2) contains supplementary material, which is available to authorized users.

## Background

Selective decontamination of the digestive tract (SDD) and selective oropharyngeal decontamination (SOD) are prophylactic antibiotic interventions for patients in intensive care units (ICUs). They consist of enteral application of non-absorbable antimicrobial agents, most often amphotericin B, tobramycin and colistin, aiming to eradicate yeasts, *Staphylococcus aureus* and (facultative) aerobic Gram-negative bacteria. SDD includes topical antibiotics applied daily to the mouth and the stomach during the whole ICU admission, in combination with a short initial course of intravenous antibiotics. SOD only applies antibiotics to the mouth. Use of SDD or SOD has been shown to reduce the incidence of ventilator-associated pneumonia (VAP) and to improve patient survival [1–5].

SDD and SOD imply daily administration of topical antibiotics during ICU stay. Prolonged use of antibiotics is generally associated with an increased risk of antibiotic resistance, especially in critically ill ICU patients. Yet, SDD and SOD have not been associated with increased resistance of bacteria colonizing the digestive tract [2, 3, 6, 7]. In fact, in recent large studies from the Netherlands, antibiotic resistance in Gram-negative bacteria colonizing the digestive tract was reduced during SOD and even more during SDD [2, 3]**.** Possible, but unproven explanations for this decrease in resistance during use of SDD and SOD include decreased incidence of nosocomial infections with a concomitant decrease in the use of systemic antibiotics during SDD and SOD, very high enteral concentrations of tobramycin and colistin that may even eradicate or suppress aerobic Gram-negative bacteria classified as resistant based on the determined minimal inhibitory concentrations [6], or masking the presence of resistant bacteria in culture medium due to the topical antibiotics present in fecal samples [8]. If topical antibiotics in the intestinal tract suppress bacterial growth without eradication or if these antibiotics result in false-negative culture results, rapid emergence of resistant bacteria after discontinuation of SDD, usually after patient discharge from ICU, could be expected.

So far, all analyses on the effects of SDD and SOD on antibiotic resistance have been restricted to the period of administration of prophylactic antibiotics. Debate continues on the effects of SDD and SOD on the emergence of antibiotic resistance [5]. We, therefore, quantified the acquisition rates of rectal recolonization with resistant aerobic Gram-negative bacteria in patients during the first 10 days after discharge from ICU and discontinuation of SDD or SOD.

### Patients

This trial has been registered in the Netherlands Trial Registry (number NTR3311). It was a prospective, observational study in medical and surgical ICU patients in three teaching hospitals in the Netherlands (Academic Medical Center, Amsterdam, University Medical Center, Utrecht, and Leiden University Medical Center, Leiden), performed in parallel to a Dutch 16-center cluster-randomized trial evaluating the effects of SDD and SOD on colonization with antibiotic resistant Gram-negative bacteria in respiratory and perineal samples. In this trial, performed between August, 2009 and February, 2013 ICUs crossed over from using SDD to SOD or from SOD to SDD in all patients with an expected ICU length of stay > 48 h [9]. When crossing over from SDD to SOD or from SOD to SDD there was a 1-month washout period during which no patients were included in thisstudy.

From September 2010 to April 2013, all patients discharged from the ICUs of three hospitals were eligible for inclusion if they had received SDD or SOD treatment for more than 4 days. Exclusion criteria were age younger than 18 years, and treatment with enteral antibiotics other than SDD or SOD during ICU stay. If patients had been admitted to ICU more than one time during hospital admission, only the hospitalization period after the last ICU admission was eligible.

## Methods

The SDD regimen was identical to that used in previous trials and consisted of oropharyngeal application (every 6 h) of approximately 0.5 g of a paste containing colistin, tobramycin and amphotericin B each in a 2% concentration. In patients with a tracheostomy the same paste was applied around the tracheostomy. In addition, a 10 ml suspension containing 100 mg colistin, 122 mg tobramycin and 500 mg amphotericin B was given via the nasogastric tube four times daily until ICU discharge. In patients with a duodenal tube or jejunostomy, 5 ml of the suspension was given via the gastric tube and the remaining 5 ml via the duodenal tube or jejunostomy. Patients with colostomy or ileostomy received SDD suppositories (containing 100 mg colistin, 61 mg tobramycin and 200 mg amphotericin B) twice daily in the distal part of the gut. In addition, cefotaxime (1000 mg, every 6 h) was administered intravenously during the first 4 days. Other intravenous antibiotics were administered therapeutically if clinically indicated. The SOD regimen consisted of oropharyngeal application of the same paste as used for SDD. In patients with tracheostomy the paste was also applied around the tracheostomy. No other prophylactic antibiotics were administered. Intravenous antibiotics were given only if clinically indicated.

Rectal colonization with any resistant aerobic Gram-negative bacteria was determined by rectal swab cultures at time points 0, 3, 6 and 10 days after discharge from the ICU. Samples were screened for presence of resistant aerobic Gram-negative bacteria by selective media (ChromID ESBL agar; McConkey agar with tobramycin 8 mg/L and McConkey agar with ciprofloxacin at 2 mg/L) (bioMérieux Benelux B.V., Zaltbommel, The Netherlands). Isolates were identified by the use of matrix-assisted laser desorption ionization-time of flight mass spectrometry (MALDI BioTyper, Bruker Daltonik GmbH, Leipzig, Germany) according to the manufacturer’s instructions.

Susceptibility tests were performed using the Vitek2 system (bioMérieux Benelux B.V., Zaltbommel, The Netherlands). Antimicrobial susceptibility results were interpreted according to the EUCAST guidelines (v1.1, v 1.2 from August 2011 and v1.3 from January 2012).

In accordance with an earlier study [3], multiresistance pattern A was defined as resistance to tobramycin and to ciprofloxacin or ceftazidime. Multiresistance pattern B was resistance to tobramycin, to ciprofloxacin and to ceftazidime.

Gram-negative microorganisms intrinsically non-susceptible to applied antibiotics were excluded from the analysis: *Achromobacter spp*. and *Stenotrophomonas spp*. for all tested antibiotics; *Enterobacter spp*. for ceftazidime; *Hafnia alvei* for ceftazidime; *Morganella spp*. for colistin and ceftazidime; *Proteus mirabilis* for colistin; *Proteus vulgaris* for colistin and ceftazidime; *Providentia spp.* for aminoglycosides and ceftazidime; *Serratia spp*. for colistin and ceftazidime; *Citrobacter* for ceftazidime.

In a post-hoc analysis we also determined rectal carriage rates with all antibiotic-resistant Gram-negative bacteria (ARGNB), including bacteria intrinsically non-susceptible to the applied antibiotics.

### Statistical analysis

The primary endpoint was time to first colonization with any Gram-negative bacteria resistant to tobramycin, ceftazidime, ciprofloxacin, meropenem or colistin that was not present at time of ICU discharge. Time to new colonization with resistant bacteria was compared between the regimes by Cox proportional hazards modeling with adjustment for hospital as a covariate. Differences in the proportion of patients colonized with resistant bacteria were tested by chi-square analysis. Based on earlier studies, we estimated that 10% of patients would be colonized within 10 days after ICU discharge with any resistant aerobic Gram-negative bacteria [3]: 620 patients per study group were required to exclude the hypothesis that colonization with any resistant strain differs more than 5% between groups (two-sided,alpha 0.05, beta 0.20).

## Results

### Patient characteristics

During the study period, 1370 patients were discharged from the ICU to a medical or surgical hospital ward, and rectal swabs had been obtained from 996 patients (507 during SDD and 489 during SOD) at the day of ICU discharge. Patients in the SOD group more often had planned ICU admission (22% vs. 16%). Apart from differences in referring medical specialty, all other patient characteristics were comparable (Table 1). Mean ICU length of stay was 13.4 ± 13.8 days in patients treated with SOD and 12.8 ± 12.4 days in patients treated with SDD (*p* = 0.52), and 87.9% and 87.9% (SDD and SOD, respectively) of the patients survived the hospital stay.

**Table 1:** Baseline characteristics

	SOD (*n* = 489)	SDD (*n* = 507)	*p* value
Characteristic			
Mean age (mean + SD) (years)	59.5 + 15.6	59.6 + 15.3	0.93
Male (%)	61.0	63.3	0.51
Planned ICU admission (%)	22.2	16.1	0.03
Surgical	47.0	48.6	0.18
Medical	53.0	51.4	0.18
Apache IV score	75.9 ± 26.3	73.2 ± 27.3	0.22
Referring speciality (%)			
Cardiology	8.6	7.3	0.92
Internal medicine	13.6	9.4	0.19
Pulmonology	3.2	4.3	0.30
Neurology	5.8	10.0	0.01
Surgery	20.0	19.0	0.73
Thoracic surgery	27.4	17.7	0.01
Neurosurgery	7.8	10.4	0.07
Other	13.6	21.9	0.01
Mechanical ventilation (%)	90.5	90.7	0.96
CPR before ICU admission	9.0	11.6	0.24
Acute renal failure	9.2	7.9	0.56
Stroke	5.7	9.3	0.06
Confirmed infection at ICU admission	27.1	31.0	0.23
COPD	7.7	9.1	0.54
Diabetes mellitus	11.5	15.4	0.11
Chronic renal failure	5.3	7.7	0.19
Chronic dialysis	1.5	1.2	0.85
Metastasized cancer	2.4	1.9	0.73
Cirrhosis	1.5	0.9	0.61
Immune deficiency	10.5	9.8	0.96
Hematologic cancer	4.0	3.7	0.99
Heart failure III-IV/IV NYHA	7.5	6.1	0.48
Resp. failure III-IV/IV NYHA	5.9	5.1	0.70
ICU length of stay (days)	13.4 ± 13.8	12.8 ± 12.4	0.52
Hospital survival (%)	87.9	87.9	0.92

*SDD* selective decontamination of the digestive tract, *SOD* selective oropharyngeal decontamination, *APACHE* Acute Physiology and Chronic Health Evaluation, *CPR* cardiopulmonary resuscitation, *COPD* chronic obstructive pulmonary disease, *NYHA* New York Heart Association, *Resp.* respiratory

### Resistance prevalence at ICU discharge

Rectal colonization with a Gram-negative bacterium resistant to any of the phenotypes investigated at the time of ICU discharge in the three hospitals ranged from 3.4% to 5.2% after SDD and from 10.7% to 30.6% after SOD, yielding pooled estimates of 4.1% and 17.8% after SDD and SOD, respectively (*p* < 0.0001) (Table 2). For individual antibiotics, prevalence of resistance was highest for ceftazidime (9.4% with SOD versus 1.8% with SDD, *p* < 0.0001). Carriage of extended spectrum beta-lactamase (ESBL)-producing bacteria was demonstrated in 12.3% of patients treated with SOD and 1.4% of patients treated with SDD (*p* < 0.0001). Prevalence of resistance against meropenem was 0.6% with SOD and 0% with SDD (*p* = 0.23) and against colistin it was 2.0% with SOD and 1.0% with SDD (*p* = 0.27). Detailed data about resistance phenotype for different bacteria are given in Additional file 1. In post-hoc analysis the prevalence of rectal colonization with any ARGNB, including intrinsically resistant bacteria, at ICU discharge was 4.9% after SDD and 22.3% after SOD (*p* < 0.0001) (Additional file 2).

**Table 2:** Rectal colonization with resistant Gram-negative bacteria at and after ICU discharge and number of resistance phenotypes (to ceftazidime, tobramycin, colistin, meropenem and ciprofloxacin)

	Number of patients colonized with ARGNB at ICU discharge			Number of patients acquiring colonization with ARGNB after ICU discharge		
	SDD (*n* = 507)	SOD (*n* = 489)	*p* value*	SDD (*n* = 426)	SOD (*n* = 409)	*p* value*
At least one AR-GNB						
ICU A	8/154 (5.2%)	29/271 (10.7%)	0.05	14/134 (10.4%)	38/224 (17.0%)	0.09
ICU B	6/115 (5.2%)	38/124 (30.6%)	< 0.0001	9/95 (9.5%)	16/96 (16.7%)	0.14
ICU C	8/238 (3.4%)	20/94 (21.3%)	< 0.0001	18/197 (9.1%)	10/89 (11.2%)	0.6
All	22/507 (4.3%)	87/489 (17.8%)	< 0.0001	41/426 (9.6%)	64/409 (15.6%)	0.01
Different ARGNB	23	97	< 0.0001	66	90	0.02
*E.coli*	10	46		20	36	
*Enterobacter sp*.	1	11		8	15	
*K. pneumoniae*	1	9		6	4	
*P. aeruginosa*	3	15		6	15	
other	8	16		26	20	
Resistance phenotypes						
Ceftazidime	9	46		21	27	
Ciprofloxacin	11	40		12	29	
Tobramycin	17	33		23	36	
Meropenem	0	3		1	3	
Colistin	5	10		7	11	
ESBL	7	60		20	24	

*ARGNB* antibiotic resistant Gram-negative bacteria, *SDD* selective decontamination of the digestive tract, *SOD* selective oropharyngeal decontamination, *ESBL* extended spectrum beta-lactamase

*p value by Chi square analysis for difference between SOD and SDD

### Acquisition of resistance after ICU discharge

**Fig. 1:**
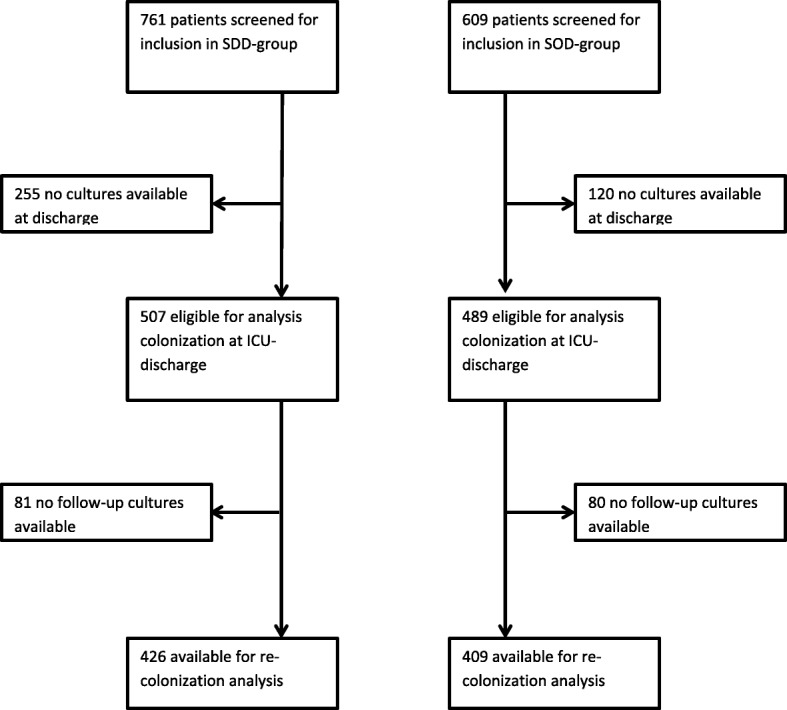
Trial profile. SDD, selective decontamination of the digestive tract; SOD, selective oropharyngeal decontamination

Occurrence of newly acquired colonization was studied in 835 patients (426 treated with SDD and 409 treated with SOD), with carriage determined at ICU discharge and at least one successive time point thereafter (Fig. 1). The mean follow up of these patients was 7.9 ± 3.9 in the SDD and 7.5 ± 4.0 days in the SOD group. The mean number of rectal swabs obtained per patient was 3.1 ± 0.9 after SDD and 3.3 ± 1.0 after SOD.

**Fig. 2:**
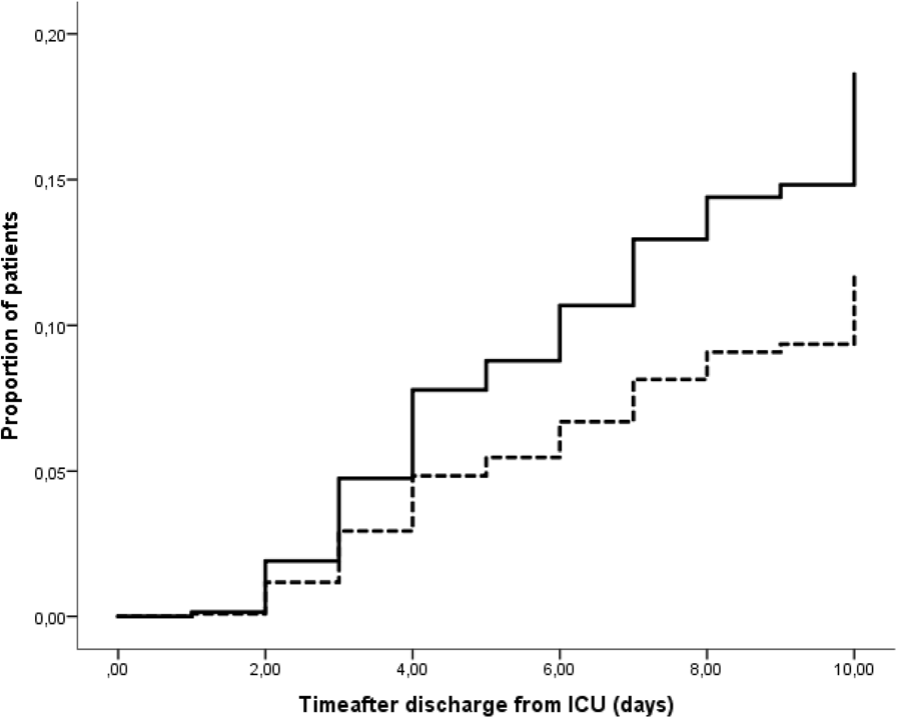
Time to first colonization with any Gram-negative bacteria resistant to tobramycin, ceftazidime, ciprofloxacin, meropenem or colistin in patients who were not already colonized with that specific resistant Gram-negative bacteria at time of ICU discharge. Bacteria that are intrinsically resistant to the antibiotics are excluded. Analysis after adjustment for individual ICU. *p* = 0.02 by Cox regression analysis for the difference between patients treated with selective decontamination of the digestive tract (dashed line; *n* = 426) or selective oropharyngeal decontamination (solid line; *n* = 409)

**Fig. 3:**
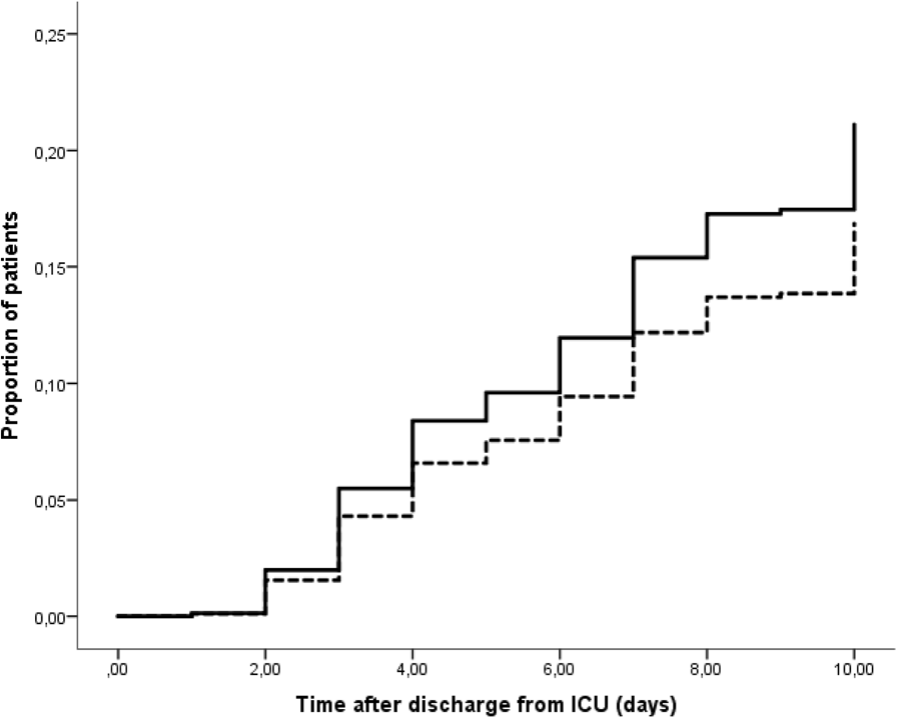
Additional analysis on all antibiotic-resistant Gram-negative bacteria (ARGNB), including intrinsically resistant bacteria. Time to first colonization with any Gram-negative bacteria resistant to tobramycin, ceftazidime, ciprofloxacin, meropenem or colistin in patients who were not already colonized with that specific resistant Gram-negative bacteria at time of ICU discharge. Analysis after adjustment for individual ICU. *p* = 0.16 by Cox regression analysis for the difference between patients treated with selective decontamination of the digestive tract (dashed line; *n* = 426) or selective oropharyngeal decontamination (solid line; *n* = 409)

The primary endpoint of this study, i.e. a new episode of colonization with any Gram-negative bacteria resistant to ceftazidime, tobramycin, ciprofloxacin, meropenem or colistin was reached in 41 of 426 patients in the SDD group and in 64 of 409 patients in the SOD group (*p* = 0.01, Table 2). The mean time until colonization was 6.5 days with SDD and 4.8 days with SOD. The hazard ratio for acquiring new carriage after SDD, compared to SOD and adjusted for ICU, was 0.61 (95% CI 0.40–0.91, *p* = 0.02 by Cox regression analysis), the cumulative risks of acquisition at day 10 were 13% and 18% after SDD and SOD respectively (Fig. 2). In post-hoc analysis of all ARGNB, including intrinsically resistant bacteria, the hazard ratio for acquiring new carriage with ARGNB after SDD, compared to SOD, and adjusted for ICU, was 0.78 (95% CI 0.55–1.11, *p* = 0.16 by Cox regression analysis, Fig. 3).

For individual antibiotics, only the hazard ratio for acquiring carriage of ciprofloxacin-resistant bacteria was significantly different; for SDD, compared to SOD, 0.48, 95% CI 0.24–0.99; *p* = 0.05) (Additional file 3). Clinically relevant or statistically significant differences were not found for resistance against ceftazidime, tobramycin, meropenem, and colistin or for ESBL-producing bacteria and bacteria with multiresistance A and multiresistance B patterns (Additional file 4).

Newly acquired resistance carriage was most frequently observed for ciprofloxacin (Additional file 5; 7.1% for SOD vs. 2.8% for SDD-treated patients, *p* = 0.007) and tobramycin (8.8% for SOD and 5.4% for SDD, *p* = 0.07). The incidence of new colonization with ESBL-producing bacteria was 5.9% with SOD patients and 4.7% with SDD (*p* = 0.54). Acquired carriage with Gram-negative bacteria resistant to colistin was observed in 11 (2.7%) and 7 patients (1.6%) in the SOD and SDD groups, respectively (*p* = 0.42). It should be noted that in this analysis more than one different resistant bacteria could be cultured from individual patients. *Escherichia coli* was the most frequently cultured resistant microorganism (Table 2 and Additional file 5; results for post-hoc analysis on all ARGNB, including intrinsically resistant bacteria in Additional file 2).

In time, the prevalence of carriage with at least one ARGNB gradually increased (from 4.3% at *T* = 0 to 11.3% at day 10 after SDD, whereas the prevalence after SOD remained stable between *T* = 0 and day 6 (17.8–18.9%), but declined to 12.5% at day 10. At this time point the prevalence was no longer significantly different between both groups (*p* = 0.7) (Table 3 and Additional file 6). The cumulative number of different resistant bacteria and of resistance phenotypes to individual antibiotics (ceftazidime, ciprofloxacin, tobramycin, meropenem and colistin) at the four time points of measurement is shown in Table 3. After ICU discharge, clearance of ARGNB was comparable in both groups with 61–82% of cultured ARGNB still present at the next assessment (Additional file 7). Acquisition of new ARGNB between different time points after ICU discharge is shown in Additional file 7.

**Table 3:** Rectal colonization with resistant Gram-negative bacteria at ICU discharge and at different time points after ICU discharge and number of resistance phenotypes (to ceftazidime, tobramycin, colistin, meropenem and ciprofloxacin)

	SDD				SOD			
	*n*	Number of patients colonized with any ARGNB (%)	Number of resistant bacteria (range of different bacteria per patient)	Cumulative number of resistance phenotypes (average number per ARGNB)	*n*	Number of patients colonized with any ARGNB (%)	Number of resistant (range of different bacteria per patient)	Cumulative number of resistance phenotypes (average number per ARGNB)
ICU discharge	507	22 (4.3)	23 (1–2)	49 (2.1)	489	87 (17.8)	97 (1–2)	195 (2.0)
*E. coli*			10				46	
*Enterobacter sp.*			1				11	
*K. pneumoniae*			1				9	
*P. aeruginosa*			3				15	
other			8				16	
Day 3	262	20 (7.6)	21 (1–2)	51 (2.4)	317	63 (19.9)	73 (1–3)	145 (2.0)
*E. coli*			6				34	
*Enterobacter sp.*			3				9	
*K. pneumoniae*			1				5	
*P. aeruginosa*			2				9	
other			9				16	
Day 6	326	25 (7.7)	26 (1–2)	51 (2.0)	323	61 (18.9)	64 (1–2)	124 (1.9)
*E. coli*			7				39	
*Enterobacter sp.*			4				8	
*K. pneumoniae*			1				4	
*P. aeruginosa*			2				4	
other			12				9	
Day 10	230	26 (11.3)	29 (1–2)	56 (1.9)	224	28 (12.5)	32 (1–2)	61 (1.9)
*E. coli*			7				15	
*Enterobacter sp.*			1				5	
*K. pneumoniae*			3				1	
*P. aeruginosa*			4				5	
other			14				6	

Bacteria may have been present at ICU discharge or acquired after ICU discharge

*ARGNB* antibiotic-resistant Gram-negative bacteria

## Discussion

In this study in 996 patients, prevalence of intestinal carriage with antibiotic-resistant Gram-negative bacteria at the time of ICU discharge was higher in patients treated with SOD, compared to patients who had received SDD during the ICU-stay, as were acquisition rates with such bacteria after ICU discharge. Yet, 10 days after ICU discharge the prevalence of intestinal carriage with antibiotic-resistant Gram-negative bacteria was comparable in both patient groups.

In the SDD and SOD groups, acquisition rates of resistant bacteria, not detected at the time of ICU discharge, were 13 and 18%, respectively. We are not aware of similar studies quantifying colonization acquisition after ICU discharge. It is, therefore, not possible to establish a causal relationship between SDD or SOD and these acquisitions, and the observations may also reflect the carriage dynamics after ICU discharge, irrespective of administration of prophylactic enteral antibiotics.

Our findings are in accordance with previous studies, showing that intestinal carriage with antibiotic resistant Gram-negative bacteria was lower during SDD, compared to SOD or standard care [2, 3, 9]. In the only study that systematically monitored intestinal colonization with any Gram-negative bacteria (resistant or non-resistant) during SOD, carriage was similar in ICU patients that had received SOD and patients that had received standard care without enteral antibiotics [10]. In that study, SOD, compared to placebo, was also not associated with differences in bacterial carriage in gastric aspirates, strongly suggesting that SOD does not modulate the intestinal flora. Moreover, in unit-wide point-prevalence surveys intestinal carriage with antibiotic-resistant Gram-negative bacteria was similar with SOD compared to standard care [3].

This difference in carriage of ARGNBs at the time of ICU discharge could result from true eradication or suppression of Gram-negative bacteria or from false-negative culture results due to the presence of antibiotics in material obtained for culture. The latter two mechanisms, true suppression or false-negative cultures, both reflect the presence of bacteria, yet in quantity not surpassing the detection limit of semi-quantitative culture methods. Discontinuation of antibiotic administration, which occurs after ICU discharge, could then lead to rapid “acquisition”.

Indeed, early acquisition of colonization was demonstrated within the first few days after ICU discharge, but this tended to occur more frequently in patients that had received SOD, compared to SDD. This finding does not support the hypothesis that lower carriage at the time of ICU discharge resulted from suppression or false-negative culture results, although it can not fully be excluded that the enterally administered antibiotics during SDD are present in the 10-day follow-up period, still suppressing growth of ARGNB from rectal samples. Unfortunately, we have no data on antibiotic concentrations in stools after ICU discharge.

The prevalence of carriage with antibiotic-resistant bacteria in general hospital wards is low in Dutch hospitals. It is unlikely that treated with SOD were exposed to higher colonization pressures after ICU discharge. Patients were discharged to specialty wards depending on bed availability and there was no difference in discharge policy between study periods. As sample collection was restricted to the included patients the role of cross-colonization in these acquisition events cannot be determined.

Despite the lower carriage at the time of ICU discharge and the lower hazard for acquiring carriage with resistant Gram-negative bacteria for SDD patients, carriage rates at day 10 after ICU discharge were comparable with SDD and SOD. This resulted from gradually increasing proportions of patients treated with SDD with carriage, and stable prevalence until day 6 followed by a decline in prevalence at day 10 in patients treated with SOD. The higher disappearance rate of resistant bacteria in patients with the highest prevalence of ARGNBs may suggest first-order kinetics, i.e. a fixed proportion of resistant bacteria colonizing patients disappears per unit of time. Absolute disappearance is then more likely to occur in patients treated with SOD, as they start with a higher prevalence. Indeed, we found that persistence of cultured ARGNB in the next sample was 62–82% in both groups. The rapid clearance of resistant bacteria is in contrast with findings by Haverkate and co-authors [11]. They reported that the mean time to clearance of highly resistant enterobacteriaceae was 1.4 months. However, whereas we studied all patients discharged from the ICU, Haverkate and co-authors only included patients if they were readmitted to the same ICU, potentially resulting in a selected population with a different case mix of patients.

Our findings provide new - observational - insights into the intestinal colonization dynamics of ICU patients that have received SDD or SOD. These dynamics are still largely unstudied, and thus unexplained. With next-generation sequencing approaches a marked increase in aminoglycoside-resistance genes was demonstrated in 12 ICU patients all treated with SDD, during the course of critical illness [12]. Yet, whether this increase can be attributed to SDD remains to be determined, as the intestinal resistome of ICU patients not treated with SDD have not been studied. Our finding of rapid acquisition of resistant bacteria after discharge from the ICU and discontinuation of SDD/SOD could be compatible with a scenario in which resistance genes accumulate in the non-culturable flora during the ICU stay and antibiotic exposure, followed by recolonization with commensal - antibiotic susceptible - Gram-negative bacteria after discontinuation of antibiotics in SDD/SOD with subsequent transfer of resistance genes from non-pathogenic anaerobic bacteria to potentially pathogenic bacteria colonizing the gut. Further detailed studies on the dynamics of the intestinal resistome are warranted.

In accordance with earlier studies, the predefined objective of our study was rectal colonization with ARGNB, excluding bacteria that are intrinsically non-susceptible to certain antibiotics, e.g. *Enterobacter cloacae*, resistant to ceftazidime. Indeed, these bacteria can not acquire resistance against these antibiotics due to treatment with SDD/SOD, but antibiotic prophylaxis might also select for bacteria that are intrinsically non-susceptible. We, therefore, performed a post-hoc analysis of all ARGNB, including bacteria that are intrinsically resistant. Consequently, colonization prevalence with ARGNB was higher at each time point, mostly by an increase in *Morganella sp*., *Citrobacter sp*. and *Enterobacter sp*. In this post-hoc analysis, colonization at ICU discharge was lower after SDD compared to SOD, but the difference in acquisition rates between SDD and SOD after ICU discharge were similar and no longer statiscally significant.

There are several study limitations. First, for feasibility reasons, the period of follow up was limited to 10 days after ICU discharge and cessation of SDD or SOD. Despite the study size, the study was underpowered to quantify effects on infections with ARGNB. Furthermore, as the study was performed in three tertiary care centers we cannot rule out that effects on early recolonization are different in settings with other patient populations. Our study was restricted to intestinal carriage with antibiotic-resistant Gram-negative bacteria. In a previous study acquisition of respiratory tract carriage with antibiotic-resistant Gram-negative bacteria also occurred less frequently in patients that received SDD, compared to patients receiving SOD or standard care [13]. Further studies are needed to elucidate the mechanisms underlying those observations.

Our study was performed in three Dutch hospitals, all considered to have low prevalence of antibiotic resistance, compared to most other countries, which reduces the generalizability of our findings. How these findings can be extrapolated to areas with high endemicity of (multi)resistant Gram-negative strains remains uncertain. Recently, a multicenter cluster-randomized trial of decontamination strategies in 13 European ICUs has been completed. Findings from this trial are not yet available but will provide more information on the use of SDD and SOD in areas with higher prevalence of resistance (N.L. Plantinga and B.H. Wittekamp; personal communication). A prospective controlled study performed in an area with a high level of carbapenem-resistant Enterobacteriaceae (CRE) showed that non-absorbable drugs eradicated CRE gastrointestinal colonization significantly better than spontaneous eradication [14]. In contrast, a retrospective analysis performed in a Netherlands ICU reported an increase of colistin-resistant Enterobacteriacaeae after the introduction of SDD [15]. More research on the effects of SDD and SOD on infections and mortality and on antibiotic resistance in those areas is needed. Finally, we did not test for recolonization with methicillin-resistant *Staphylococcus aureus* (MRSA) and vancomycin-resistant *Enterococci* (VRE), due to the very low prevalence in our country [16].

## Conclusions

The rate of colonization with resistant Gram-negative bacteria (excluding intrinsically resistant bacteria) during ICU stay is lower in patients using SDD compared to those using SOD. However, colonization 10 days after ICU discharge was comparable in both groups (13–18%). This high and rapid acquisition, which was most pronounced after SOD, has not been shown before. At the same time, the clearance of resistant bacteria after ICU discharge is higher after SOD leading to comparable carriage of resistance at 10 days after ICU discharge. This rapid clearance of resistance is a new finding and more studies are needed to better understand the complexity of the dynamics of colonization with resistant bacteria in ICU patients. In post-hoc analysis of all ARGNB, including intrinsically resistant bacteria, colonization at ICU discharge was lower after SDD, but the difference in acquisition rates between SDD and SOD after ICU discharge were similar and no longer statistically significant.

## Additional files

Additional file 1: **Table S1.** Colonization with resistant Gram-negative bacteria at ICU discharge. (DOCX 17 kb)

Additional file 2: **Table S2.** Analysis in which intrinsically resistant bacteria are also included. Rectal colonization with resistant Gram-negative bacteria at and after ICU discharge. (DOCX 39 kb)

Additional file 3: **Figure S1.** Time to first rectal colonization after ICU discharge with Gram-negative bacteria resistant to ciprofloxacin. (DOCX 20 kb)

Additional file 4: **Figure S2-S8.** Time to first rectal colonization after ICU discharge with Gram-negative bacteria resistant to ceftazidime, tobramycin, meropenem, colistin, ESBL-producing bacteria and bacteria with multiresistance pattern A or B. (DOCX 72 kb)

Additional file 5: **Table S3.** Colonization with resistant Gram-negative bacteria after ICU discharge. (DOCX 17 kb)

Additional file 6: **Table S4.** Analysis in which intrinsically resistant bacteria are also included. Rectal colonization with resistant Gram-negative bacteria at ICU discharge and at different time points after ICU discharge. (DOCX 38 kb)

Additional file 7: **Table S5.** Rectal colonization, acquisition and persistence of resistant Gram-negative bacteria (ARGNB) at ICU discharge and at *T* = 3, 6 and 10 days. (DOCX 15 kb)

## Abbreviations

ARGNB: Antibiotic-resistant Gram-negative bacteria
CRE: Carbapenem-resistant enterobacteriaceae
ESBL: Extended spectrum beta-lactamase
ICU: Intensive care unit
MRSA: Methicillin-resistant *Staphylococcus aureus*
SDD: Selective decontamination of the digestive tract
SOD: Selective oropharyngeal decontamination
VAP: Ventilator-associated pneumonia
VRE: Vancomycin-resistant enterococcus

## References

[1] Plantinga NL, de Smet A, Oostdijk EAN, de Jonge E, Camus C, Krueger WA, Bergmans D, Reitsma JB, Bonten MJM (2017). Selective digestive and oropharyngeal decontamination in medical and surgical ICU patients: individual patient data meta-analysis. Clin Microbiol Infect.

[2] de Jonge E, Schultz MJ, Spanjaard L, Bossuyt PM, Vroom MB, Dankert J, Kesecioglu J (2003). Effects of selective decontamination of digestive tract on mortality and acquisition of resistant bacteria in intensive care: a randomised controlled trial. Lancet.

[3] de Smet AM, Kluytmans JA, Cooper BS, Mascini EM, Benus RF, van der Werf TS, van der Hoeven JG, Pickkers P, Bogaers-Hofman D, van der Meer NJ (2009). Decontamination of the digestive tract and oropharynx in ICU patients. N Engl J Med.

[4] Krueger WA, Lenhart FP, Neeser G, Ruckdeschel G, Schreckhase H, Eissner HJ, Forst H, Eckart J, Peter K, Unertl KE (2002). Influence of combined intravenous and topical antibiotic prophylaxis on the incidence of infections, organ dysfunctions, and mortality in critically ill surgical patients: a prospective, stratified, randomized, double-blind, placebo-controlled clinical trial. Am J Respir Crit Care Med.

[5] Vincent JL, Jacobs F (2011). Effect of selective decontamination on antibiotic resistance. Lancet Infect Dis.

[6] de Jonge E (2005). Effects of selective decontamination of digestive tract on mortality and antibiotic resistance in the intensive-care unit. Curr Opin Crit Care.

[7] Silvestri L, van Saene HK, Casarin A, Berlot G, Gullo A (2008). Impact of selective decontamination of the digestive tract on carriage and infection due to gram-negative and gram-positive bacteria: a systematic review of randomised controlled trials. Anaesth Intensive Care.

[8] Verbrugh HA (2003). Selective decontamination of digestive tract in intensive care. Lancet.

[9] Oostdijk EAN, Kesecioglu J, Schultz MJ, Visser CE, de Jonge E, van Essen EHR, Bernards AT, Purmer I, Brimicombe R, Bergmans D (2014). Effects of decontamination of the oropharynx and intestinal tract on antibiotic resistance in ICUs: a randomized clinical trial. JAMA.

[10] Bergmans DC, Bonten MJ, Gaillard CA, Paling JC, van der Geest S, van Tiel FH, Beysens AJ, de Leeuw PW, Stobberingh EE (2001). Prevention of ventilator-associated pneumonia by oral decontamination: a prospective, randomized, double-blind, placebo-controlled study. Am J Respir Crit Care Med.

[11] Haverkate MR, Derde LP, Brun-Buisson C, Bonten MJ, Bootsma MC (2014). Duration of colonization with antimicrobial-resistant bacteria after ICU discharge. Intensive Care Med.

[12] Buelow E, Gonzalez TB, Versluis D, Oostdijk EA, Ogilvie LA, van Mourik MS, Oosterink E, van Passel MW, Smidt H, D'Andrea MM (2014). Effects of selective digestive decontamination (SDD) on the gut resistome. J Antimicrob Chemother.

[13] de Smet AM, Kluytmans JA, Blok HE, Mascini EM, Benus RF, Bernards AT, Kuijper EJ, Leverstein-van Hall MA, Jansz AR, de Jongh BM (2011). Selective digestive tract decontamination and selective oropharyngeal decontamination and antibiotic resistance in patients in intensive-care units: an open-label, clustered group-randomised, crossover study. Lancet Infect Dis.

[14] Oren I, Sprecher H, Finkelstein R, Hadad S, Neuberger A, Hussein K, Raz-Pasteur A, Lavi N, Saad E, Henig I (2013). Eradication of carbapenem-resistant Enterobacteriaceae gastrointestinal colonization with nonabsorbable oral antibiotic treatment: a prospective controlled trial. Am J Infect Control.

[15] Halaby T, Al Naiemi N, Kluytmans J, van der Palen J, Vandenbroucke-Grauls CM (2013). Emergence of colistin resistance in Enterobacteriaceae after the introduction of selective digestive tract decontamination in an intensive care unit. Antimicrob Agents Chemother.

[16] NethMap. Consumption of antimicrobial agents and antimicrobial resistance among medically important bacteria in the Netherlands in 2015. http://www.swab.nl/swab/cms3.nsf/uploads/8E8465E1538C90ADC125802F002DFC4E/$FILE/rapport%202016-0060%20Nethmap%20Maran%202016%20met%20erratum%20beveiligd.pdf.

