# Supplementary material for: Carriage of antibiotic-resistant Gram-negative bacteria after discontinuation of selective decontamination of the digestive tract (SDD) or selective oropharyngeal decontamination (SOD)
Source: Crit Care. 2018 Sep 29;22:243. doi: 10.1186/s13054-018-2170-2 (PMC6162962; doi:10.1186/s13054-018-2170-2)
Supplement: Supplementary file 1 — Table S1. Colonization with resistant Gram-negative bacteria at ICU discharge. (DOCX 17 kb) [file 13054_2018_2170_MOESM1_ESM.docx]

Additional file 1

|  | Ceftazidime | | Ciprofloxacin | | Tobramycin | | Meropenem | | Colistin | | MRA | | MRB | | ESBL | |
| --- | --- | --- | --- | --- | --- | --- | --- | --- | --- | --- | --- | --- | --- | --- | --- | --- |
|  | SDD | SOD | SDD | SOD | SDD | SOD | SDD | SOD | SDD | SOD | SDD | SOD | SDD | SOD | SDD | SOD |
| n | 507 | 489 | 507 | 489 | 507 | 489 | 507 | 489 | 507 | 489 | 507 | 489 | 507 | 489 | 507 | 489 |
| *Acinetobacter sp.* |  | 1 |  |  |  |  |  |  |  |  |  |  |  |  |  |  |
| *Citrobacter sp.* |  |  | 1 |  | 1 |  |  |  | 1 |  | 1 |  | 1 |  |  | 5 |
| *E.coli* | 4 | 19 | 8 | 25 | 7 | 15 |  | 1 | 3 | 2 | 5 | 10 | 4 | 7 | 3 | 29 |
| *Enterobacter sp.* |  |  |  | 3 |  | 2 |  |  |  | 3 |  | 2 |  | 1 | 1 | 8 |
| *Hafnia alvei* |  |  |  |  |  |  |  |  |  | 3 |  |  |  |  |  | 1 |
| *K. oxytoca* | 1 | 2 |  | 1 | 1 | 2 |  |  |  | 1 |  | 2 |  | 1 |  | 3 |
| *K. pneumoniae* | 1 | 8 |  | 6 | 1 | 5 |  |  |  | 1 | 1 | 6 |  | 5 | 1 | 8 |
| *Morganella sp.* |  |  |  | 1 | 3 | 1 |  |  |  |  | 2 | 1 |  | 1 | 2 | 1 |
| *P.aeruginosa* | 2 | 15 | 1 | 4 | 2 | 6 |  | 2 | 1 |  | 2 | 6 | 1 | 2 |  | 3 |
| *P.mirabilis* | 1 |  | 1 |  | 1 |  |  |  |  |  | 1 |  |  |  |  |  |
| *P.vulgaris* |  |  |  |  |  |  |  |  |  |  |  |  |  |  |  |  |
| *Providentia sp.* |  |  |  |  |  |  |  |  |  |  |  |  |  |  |  |  |
| *Raoultella sp.* |  | 1 |  |  |  | 1 |  |  |  |  |  | 1 |  |  |  | 1 |
| *Salmonella sp.* |  |  |  |  |  |  |  |  |  |  |  |  |  |  |  |  |
| *Serratia sp.* |  |  |  |  | 1 | 1 |  |  |  |  |  |  |  |  |  | 1 |
| Total (%) | 9(1.8) | 46(9.4) | 11(2.2) | 40(8.2) | 17(3.4) | 33(6.7) |  | 3(0.6) | 5(1.0) | 10(2.0) | 12(2.4) | 28(5.7) | 6(1.2) | 17(3.5) | 7(1.4) | 60(12.3) |
| p-value* | <0.001 | | <0.001 | | 0.01 | | 0.1 | | 0.2 | | 0.001 | | 0.02 | | <0.001 | |

**Additional file 1. Colonization with resistant Gram-negative bacteria at ICU-discharge.**

MRA=Multi-resistance pattern A (aminoglycoside resistance and [ciprofloxacin resistance or ceftazidime resistance]). MRB=Multi-resistance pattern B (aminoglycoside resistance and ciprofloxacin resistance and ceftazidime resistance). ESBL=Extended Spectrum Beta-Lactamase. In individual patients more than one resistant microorganism can be present. * p – value by Chi square for difference between SOD and SDD.
