# Supplementary material for: Carriage of antibiotic-resistant Gram-negative bacteria after discontinuation of selective decontamination of the digestive tract (SDD) or selective oropharyngeal decontamination (SOD)
Source: Crit Care. 2018 Sep 29;22:243. doi: 10.1186/s13054-018-2170-2 (PMC6162962; doi:10.1186/s13054-018-2170-2)
Supplement: Supplementary file 2 — Table S2. Analysis in which intrinsically resistant bacteria are also included. Rectal colonization with resistant Gram-negative bacteria at and after ICU discharge. (DOCX 39 kb) [file 13054_2018_2170_MOESM2_ESM.docx]

|  | **No. of patients colonized with AR-GNB at ICU-discharge** | | | **No. of patients acquiring colonization with AR-GNB after ICU discharge** | | |
| --- | --- | --- | --- | --- | --- | --- |
|  | SDD (n=507) | SOD (n=489) | p-value* | SDD (n=426) | SOD (n=409) | p-value* |
| **At least one AR-GNB** |  |  |  |  |  |  |
| ICU A | 9/154 (5.8%) | 35/271 (12.9%) | 0.02 | 20/134 (14.9%) | 42/224 (18.8.0%) | 0.35 |
| ICU B | 7/115 (6.1%) | 44/124 (35.4%) | <0.0001 | 15/95 (15.8%) | 19/96 (19.8%) | 0.47 |
| ICU C | 9/238 (3.8%) | 30/94 (31.9%) | <0.0001 | 24/197 (12.2%) | 12/89 (13.5%) | 0.76 |
| All | 25/507 (4.9%) | 109/489 (22.3%) | <0.0001 | 59/426 (13.8%) | 73/409 (17.8%) | 0.11 |
| **Different AR-GNB** | 28 | 131 |  | 99 | 108 |  |
| *E.coli* | 10 | 46 |  | 20 | 36 |  |
| *Enterobacter sp*. | 2 | 17 |  | 18 | 18 |  |
| *Morganella sp.* | 4 | 11 |  | 5 | 9 |  |
| *Citrobacter sp.* | 2 | 8 |  | 7 | 7 |  |
| *K.pneumoniae* | 1 | 9 |  | 6 | 4 |  |
| *P.aeruginosa* | 3 | 15 |  | 6 | 15 |  |
| other | 6 | 25 |  | 37 | 19 |  |
| **Resistance phenotypes** |  |  |  |  |  |  |
| Ceftazidime | 18 | 76 |  | 55 | 43 |  |
| Ciprofloxacin | 12 | 44 |  | 19 | 31 |  |
| Tobramycin | 18 | 37 |  | 32 | 39 |  |
| Meropenem | 1 | 5 |  | 5 | 3 |  |
| Colistin | 13 | 34 |  | 31 | 30 |  |
| ESBL | 7 | 60 |  | 29 | 24 |  |

**Additional file 2. Analysis in which intrinsically resistant bacteria are also included. Rectal colonization with resistant Gram-negative bacteria at and after ICU discharge and number of resistance phenotypes (to ceftazidime, tobramycin, colistin, meropenem and ciprofloxacin).** AR-GNB: Antibiotic resistant Gram-negative bacteria. ESBL=Extended Spectrum Beta-Lactamase. * p – value by Chi square for difference
