# Supplementary material for: Carriage of antibiotic-resistant Gram-negative bacteria after discontinuation of selective decontamination of the digestive tract (SDD) or selective oropharyngeal decontamination (SOD)
Source: Crit Care. 2018 Sep 29;22:243. doi: 10.1186/s13054-018-2170-2 (PMC6162962; doi:10.1186/s13054-018-2170-2)
Supplement: Supplementary file 3 — Figure S1. Time to first rectal colonization after ICU discharge with Gram-negative bacteria resistant to ciprofloxacin. (DOCX 20 kb) [file 13054_2018_2170_MOESM3_ESM.docx]

Additional file 3.


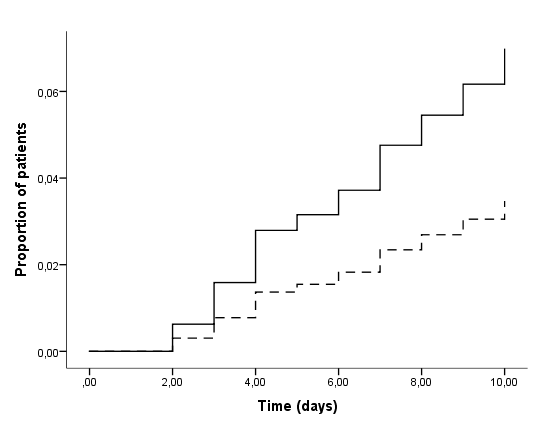


Figure S1. Time to first rectal colonization after ICU discharge with Gram-negative bacteria resistant to ciprofloxacin. Analysis after adjustment for individual ICU. P = 0·05 by Cox regression analysis for the difference between patients treated with SDD (dashed line; n=426) or SOD (solid line; n=409).
