# Supplementary material for: Carriage of antibiotic-resistant Gram-negative bacteria after discontinuation of selective decontamination of the digestive tract (SDD) or selective oropharyngeal decontamination (SOD)
Source: Crit Care. 2018 Sep 29;22:243. doi: 10.1186/s13054-018-2170-2 (PMC6162962; doi:10.1186/s13054-018-2170-2)
Supplement: Supplementary file 4 — Figure S2-S8. Time to first rectal colonization after ICU discharge with Gram-negative bacteria resistant to ceftazidime, tobramycin, meropenem, colistin, ESBL-producing bacteria and bacteria with multiresistance pattern A or B. (DOCX 72 kb) [file 13054_2018_2170_MOESM4_ESM.docx]

Additional file 4.


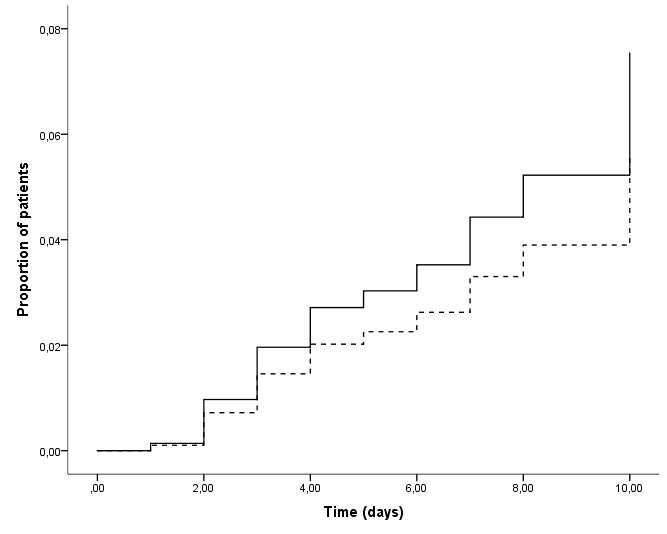


Figure S2. Time to first rectal colonization after ICU discharge with Gram-negative bacteria resistant to ceftazidime. Analysis after adjustment for individual ICU. P = 0·35 by Cox regression analysis for the difference between patients treated with SDD (dashed line; n=426) or SOD (solid line; n=409).


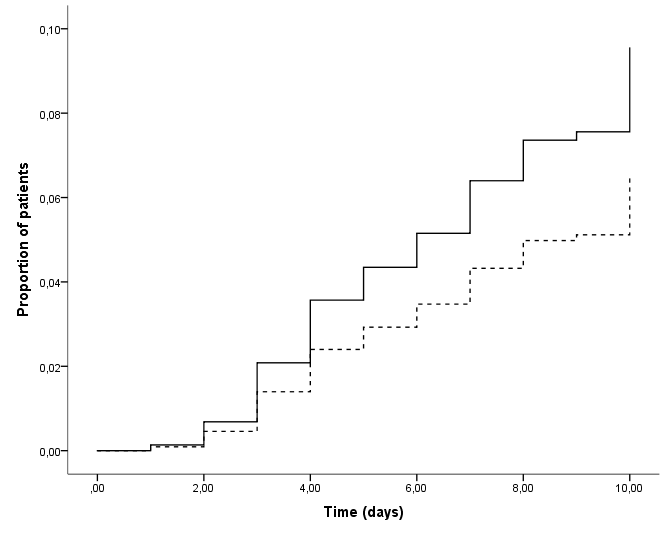


Figure S3. Time to first rectal colonization after ICU discharge with Gram-negative bacteria resistant to tobramycin. Analysis after adjustment for individual ICU. P = 0·15 by Cox regression analysis for the difference between patients treated with SDD (dashed line; n=426) or SOD (solid line; n=409).


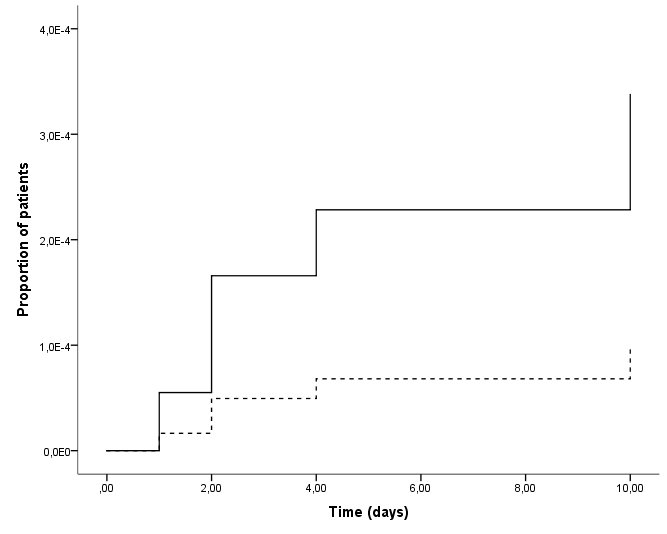


Figure S4. Time to first rectal colonization after ICU discharge with Gram-negative bacteria resistant to meropenem. Analysis after adjustment for individual ICU. P = 0·28 by Cox regression analysis for the difference between patients treated with SDD (dashed line; n=426) or SOD (solid line; n=409).


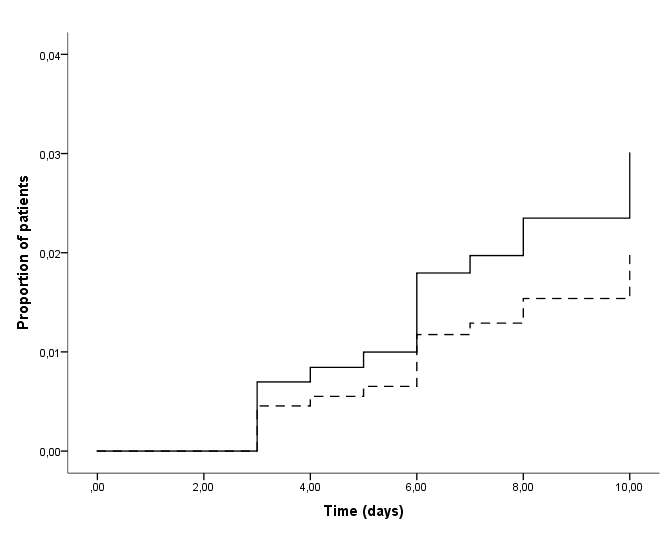


Figure S5. Time to first rectal colonization after ICU discharge with Gram-negative bacteria resistant to colistin. Analysis after adjustment for individual ICU. P = 0·40 by Cox regression analysis for the difference between patients treated with SDD (dashed line; n=426) or SOD (solid line; n=409).


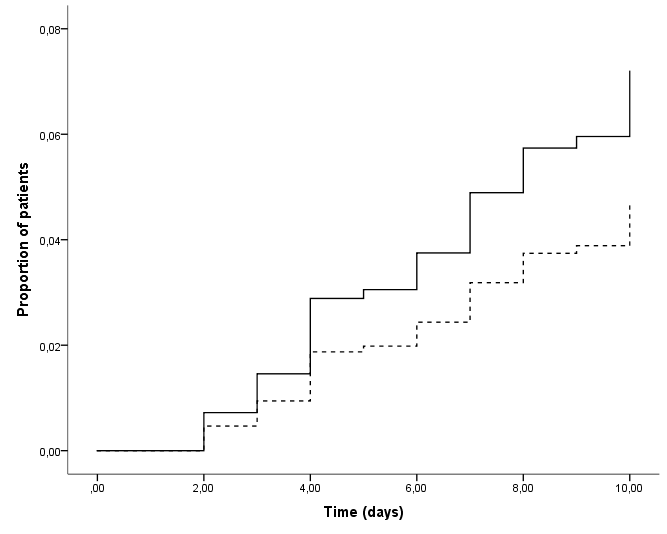


Figure S6. Time to first rectal colonization after ICU discharge with Extended Spectrum Betalactamase (ESBL) - producing Gram-negative bacteria. Analysis after adjustment for individual ICU. P = 0·19 by Cox regression analysis for the difference between patients treated with SDD (dashed line; n=426) or SOD (solid line; n=409).


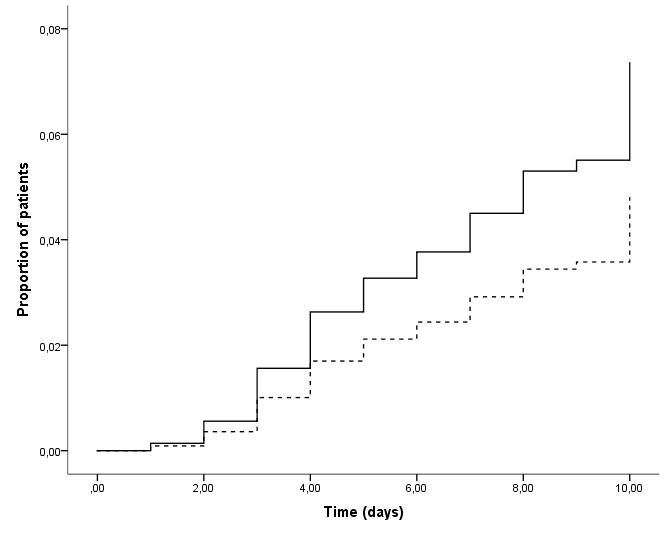


Figure S7. Time to first rectal colonization after ICU discharge with Gram-negative bacteria with Multiresistance A pattern. Analysis after adjustment for individual ICU. P = 0·18 by Cox regression analysis for the difference between patients treated with SDD (dashed line; n=426) or SOD (solid line; n=409). Multiresistance A pattern: resistant to tobramycin AND to ciprofloxacin or ceftazidime.


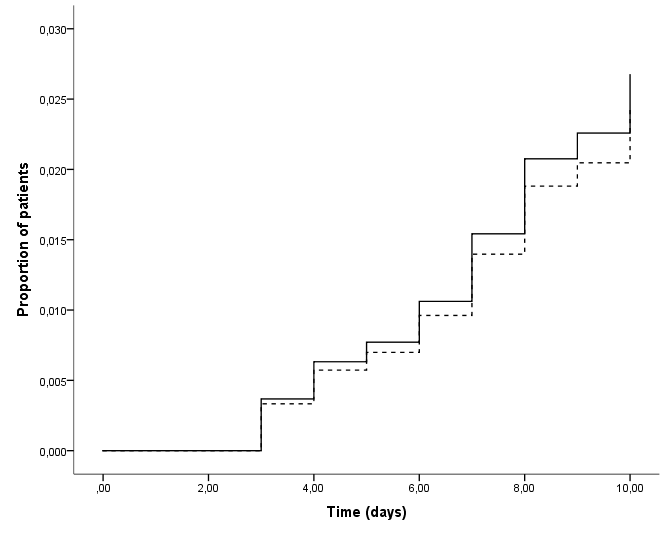


Figure S8. Time to first rectal colonization after ICU discharge with Gram-negative bacteria with Multiresistance B pattern. Analysis after adjustment for individual ICU. P = 0·84 by Cox regression analysis for the difference between patients treated with SDD (dashed line; n=426) or SOD (solid line; n=409). Multiresistance B pattern: resistant to tobramycin and to ciprofloxacin and ceftazidime.
