# Supplementary material for: Carriage of antibiotic-resistant Gram-negative bacteria after discontinuation of selective decontamination of the digestive tract (SDD) or selective oropharyngeal decontamination (SOD)
Source: Crit Care. 2018 Sep 29;22:243. doi: 10.1186/s13054-018-2170-2 (PMC6162962; doi:10.1186/s13054-018-2170-2)
Supplement: Supplementary file 5 — Table S3. Colonization with resistant Gram-negative bacteria after ICU discharge. (DOCX 17 kb) [file 13054_2018_2170_MOESM5_ESM.docx]

|  | Ceftazidime | | Ciprofloxacin | | Tobramycin | | Meropenem | | Colistin | | MRA | | MRB | | ESBL | |
| --- | --- | --- | --- | --- | --- | --- | --- | --- | --- | --- | --- | --- | --- | --- | --- | --- |
|  | SDD | SOD | SDD | SOD | SDD | SOD | SDD | SOD | SDD | SOD | SDD | SOD | SDD | SOD | SDD | SOD |
| n | 426 | 409 | 426 | 409 | 426 | 409 | 426 | 409 | 426 | 409 | 426 | 409 | 426 | 409 | 426 | 409 |
| *Acinetobacter sp.* |  | 2 |  |  |  |  |  |  |  | 1 |  |  |  |  |  |  |
| *Citrobacter sp.* |  |  |  | 1 | 2 | 2 |  |  |  |  | 1 |  |  |  |  |  |
| *E.coli* | 6 | 13 | 4 | 16 | 6 | 17 |  |  | 1 | 2 | 4 | 14 | 1 | 4 | 6 | 7 |
| *Enterobacter sp.* |  |  | 3 | 1 | 2 | 5 |  | 1 | 2 | 3 | 2 | 2 | 2 |  | 4 | 6 |
| *Hafnia alvei* |  |  |  |  |  |  |  |  | 3 |  |  |  |  |  |  |  |
| *K. oxytoca* | 4 | 2 | 1 | 2 | 2 | 2 |  |  |  | 1 | 2 | 2 | 1 |  | 2 | 1 |
| *K. pneumoniae* | 4 | 3 | 1 | 1 | 1 | 1 |  |  |  | 1 | 2 | 1 | 1 | 1 | 3 | 2 |
| *Morganella sp.* |  |  | 1 | 1 | 1 | 1 |  |  |  |  | 1 | 1 | 1 | 1 |  |  |
| *P.aeruginosa* | 4 | 6 | 1 | 5 | 2 | 5 | 1 | 2 |  | 2 | 2 | 5 | 1 | 2 |  | 3 |
| *P.mirabilis* | 2 | 1 | 1 | 2 | 4 | 2 |  |  |  |  | 2 | 1 | 1 | 1 | 2 | 2 |
| *P.vulgaris* |  |  |  |  |  |  |  |  |  |  |  |  |  |  |  |  |
| *Providentia sp.* |  |  |  |  |  |  |  |  |  | 1 |  |  |  |  |  |  |
| *Raoultella sp.* | 1 |  |  |  | 1 |  |  |  | 1 |  | 1 |  |  |  | 1 |  |
| *Salmonella sp.* |  |  |  |  |  | 1 |  |  |  |  |  |  |  |  |  |  |
| *Serratia sp.* |  |  |  |  | 2 |  |  |  |  |  |  |  |  |  | 1 |  |
| Total (%) | 21(4.9) | 27(6.6) | 12(2.8) | 29(7.1) | 23(5.4) | 36(8.8) | 1(0.3) | 3(0.7) | 7(1.6) | 11(2.7) | 17(4.0) | 26(6.4) | 8(1.9) | 9(2.2) | 20(4.7) | 24(5.9) |
| p-value* | 0.30 | | 0.004 | | 0.06 | | 0.3 | | 0.42 | | 0.16 | | 0.93 | | 0.45 | |

**Additional file 5. Colonization with resistant Gram-negative bacteria after ICU-discharge.**

Follow-up until 10 days after ICU-discharge. MRA=Multi-resistance pattern A (aminoglycoside resistance and [ciprofloxacin resistance or ceftazidime resistance]). MRB=Multi-resistance pattern B (aminoglycoside resistance and ciprofloxacin resistance and ceftazidime resistance). ESBL=Extended Spectrum Beta-Lactamase. In individual patients more than one resistant microorganism can be present. * p – value by Chi square for difference between SOD and SDD.
