# Supplementary material for: Carriage of antibiotic-resistant Gram-negative bacteria after discontinuation of selective decontamination of the digestive tract (SDD) or selective oropharyngeal decontamination (SOD)
Source: Crit Care. 2018 Sep 29;22:243. doi: 10.1186/s13054-018-2170-2 (PMC6162962; doi:10.1186/s13054-018-2170-2)
Supplement: Supplementary file 6 — Table S4. Analysis in which intrinsically resistant bacteria are also included. Rectal colonization with resistant Gram-negative bacteria at ICU discharge and at different time points after ICU discharge. (DOCX 38 kb) [file 13054_2018_2170_MOESM6_ESM.docx]

|  | SDD | | | | SOD | | | |
| --- | --- | --- | --- | --- | --- | --- | --- | --- |
|  | n | No. of patients colonized with any AR-GNB (%) | No of resistant bacteria [range of different bacteria per patient] | Cumulative number of resistance phenotypes (average no. per AR-GNB) | n | No. of patients colonized with any AR-GNB (%) | No of resistant [range of different bacteria per patient] | Cumulative number of resistance phenotypes (average no. per AR-GNB) |
| ICU discharge | 507 | 25 (4.9) | 28 [1-2] | 70 [2.5] | 489 | 109 (22.3) | 131 [1-2] | 256 [2.0] |
| *E.coli* |  |  | 10 |  |  |  | 46 |  |
| *Enterobacter sp.* |  |  | 2 |  |  |  | 17 |  |
| *K.pneumoniae* |  |  | 1 |  |  |  | 9 |  |
| *Morganella sp.* |  |  | 4 |  |  |  | 11 |  |
| *Citrobacter sp.* |  |  | 2 |  |  |  | 8 |  |
| *P.aeruginosa* |  |  | 3 |  |  |  | 15 |  |
| other |  |  | 6 |  |  |  | 25 |  |
| Day 3 | 262 | 27 (10.3) | 30 [1-2] | 77 [2.6] | 317 | 73 (23.0) | 85 [1-3] | 172 [2.0] |
| *E.coli* |  |  | 6 |  |  |  | 34 |  |
| *Enterobacter sp.* |  |  | 4 |  |  |  | 12 |  |
| *K.pneumoniae* |  |  | 1 |  |  |  | 5 |  |
| *Morganella sp.* |  |  | 2 |  |  |  | 5 |  |
| *Citrobacter sp.* |  |  | 1 |  |  |  | 6 |  |
| *P.aeruginosa* |  |  | 2 |  |  |  | 9 |  |
| other |  |  | 14 |  |  |  | 17 |  |
| Day 6 | 326 | 36 (11.0) | 39 [1-2] | 79 [2.0] | 323 | 69 (21.4) | 76 [1-2] | 144 [1.9] |
| *E.coli* |  |  | 7 |  |  |  | 39 |  |
| *Enterobacter sp.* |  |  | 7 |  |  |  | 11 |  |
| *Morganella sp.* |  |  | 4 |  |  |  | 6 |  |
| *Citrobacter sp.* |  |  | 4 |  |  |  | 4 |  |
| *K.pneumoniae* |  |  | 1 |  |  |  | 4 |  |
| *P.aeruginosa* |  |  | 2 |  |  |  | 4 |  |
| other |  |  | 14 |  |  |  | 8 |  |
| Day 10 | 230 | 33 (14.3) | 37 [1-2] | 74 [2.0] | 224 | 32 (14.3) | 39 [1-2] | 74 [1.9] |
| *E.coli* |  |  | 7 |  |  |  | 15 |  |
| *Enterobacter sp.* |  |  | 1 |  |  |  | 7 |  |
| *Morganella sp.* |  |  | 3 |  |  |  | 2 |  |
| *Citrobacter sp.* |  |  | 3 |  |  |  | 1 |  |
| *K.pneumoniae* |  |  | 3 |  |  |  | 1 |  |
| *P.aeruginosa* |  |  | 4 |  |  |  | 5 |  |
| other |  |  | 16 |  |  |  | 8 |  |

**Additional file 6. Analysis in which intrinsically resistant bacteria are also included. Rectal colonization with resistant Gram-negative bacteria at ICU discharge and at different time points after ICU discharge and number of resistance phenotypes (to ceftazidime, tobramycin, colistin, meropenem and ciprofloxacin).** Bacteria may have been present at ICU-discharge or acquired after ICU-discharge. AR-GNB: Antibiotic resistant Gram-negative bacteria.
