# Supplementary material for: Carriage of antibiotic-resistant Gram-negative bacteria after discontinuation of selective decontamination of the digestive tract (SDD) or selective oropharyngeal decontamination (SOD)
Source: Crit Care. 2018 Sep 29;22:243. doi: 10.1186/s13054-018-2170-2 (PMC6162962; doi:10.1186/s13054-018-2170-2)
Supplement: Supplementary file 7 — Table S5. Rectal colonization, acquisition and persistence of resistant Gram-negative bacteria (ARGNB) at ICU discharge and at T = 3, 6 and 10 days. (DOCX 15 kb) [file 13054_2018_2170_MOESM7_ESM.docx]

|  |  | SDD | SOD |
| --- | --- | --- | --- |
| **ICU-discharge** |  |  |  |
| *Colonization* | No. of patients | 507 | 489 |
|  | No of ARGNB | 23 (4.5%) | 97 (19.8%) |
| **Day 3** |  |  |  |
| *Colonization* | No. of patients | 262 | 317 |
|  | No of ARGNB | 21 (8.0%) | 73 (23.0%) |
| *Acquisition* | No. at risk* | 262 | 317 |
|  | New ARGNB | 12 (4.6%) | 23 (7.3%) |
| *Persistance of ARGNB present at ICU-discharge* | No. at risk** | 14 | 68 |
|  | Persistance of ARGNB present at ICU discharge | 9 (64.3%) | 46 (67.6%) |
| **Day 6** |  |  |  |
| *Colonization* | No. of patients | 326 | 323 |
|  | No of ARGNB | 26 (8.0%) | 64 (19.8%) |
| *Acquisition* | No. at risk* | 176 | 239 |
|  | New ARGNB | 10 (5.7%) | 14 (5.9%) |
| *Persistance of ARGNB present at day 3* | No. at risk** | 17 | 52 |
|  | Persistance of ARGNB present at day 3 | 12 (70.6%) | 37 (71.2%) |
| **Day 10** |  |  |  |
| *Colonization* | No. of patients | 230 | 224 |
|  | No of ARGNB | 29 (12.6%) | 32 (14.3%) |
| *Acquisition* | No. at risk* | 188 | 195 |
|  | New ARGNB | 17 (5.2%) | 16 (4.3%) |
| *Persistance of ARGNB present at day 6* | No. at risk** | 11 | 31 |
|  | Persistance of ARGNB present at day 6 | 9 (81.8%) | 19 (61.3%) |

**Additional file 7. Rectal colonization, acquisition and persistence of resistant Gram-negative bacteria (ARGNB) at ICU discharge and at T=3,6 and 10 days.** Acquisition is carriage of any ARGNB in patients who were not colonized with that same ARGNB at the previous time point. Persistance is carriage in patients who who were already colonized with same ARGNB at the previous time point. * No at risk for acquisition is number of patients with rectal cultures who were also cultured at previous time point. ** Number at risk for persistance is number of patients with rectal cultures taken who were positive for any ARGNB at previous time point.
